# The relative influence of intellectual disabilities and autism on sensory impairments and physical disability: A whole‐country cohort of 5.3 million children and adults

**DOI:** 10.1111/jar.12728

**Published:** 2020-03-18

**Authors:** Deborah Kinnear, Ewelina Rydzewska, Kirsty Dunn, Laura Hughes‐McCormack, Craig Melville, Angela Henderson, Sally‐Ann Cooper

**Affiliations:** ^1^ Mental Health and Wellbeing Group Institute of Health and Wellbeing University of Glasgow Glasgow UK

**Keywords:** autism, hearing, intellectual disabilities, observational study, physical disability, sensory impairments, vision

## Abstract

**Background:**

Intellectual disabilities and autism are lifelong and often co‐occur. Little is known on their extent of independent association with sensory impairments and physical disability.

**Methods:**

For Scotland's population, logistic regressions investigated age–gender‐adjusted odds ratios (OR) of associations, independently, of intellectual disabilities and autism with sensory impairments and physical disability.

**Results:**

1,548,819 children/youth, and 3,746,584 adults. In children/youth, the effect size of intellectual disabilities and autism, respectively, was as follows: blindness (OR = 30.12; OR = 2.63), deafness (OR = 13.98; OR = 2.31), and physical disability (OR = 43.72; OR = 5.62). For adults, the effect size of intellectual disabilities and autism, respectively, was as follows: blindness (OR = 16.89; OR = 3.29), deafness (OR = 7.47; OR = 2.36), and physical disability (OR = 6.04; OR = 3.16).

**Conclusions:**

Intellectual disabilities have greater association with the population burden of sensory impairments/physical disability, but autism is also associated regardless of overlap with intellectual disabilities. These may impact further on communication limitations due to autism and intellectual disabilities, increasing complexity of assessments/management of other health conditions. Clinicians need to be aware of these important issues.

## 1 INTRODUCTION

Having both intellectual disabilities and autism is not uncommon in children and adults. Children and adults with intellectual disabilities have markedly poorer health than other people (Carville, 2001; Cooper, Smiley, Morrison, Allan, & Williamson, 2007; Emerson & Hatton, 2007; Hughes‐McCormack et al., 2017), and this has also been reported to be the case for autistic children and adults (Croen et al., 2015; Rydzewska et al., 2018a, 2018b; Simonoff et al., 2008). However, intellectual disabilities and autism have tended to be studied separately rather than seeking the relative contribution of each condition to overall population health, and even these separate studies have largely not distinguished between those with just one or both of intellectual disabilities and autism. Of particular note is the dearth of research investigating the extent to which intellectual disabilities and autism are independently associated with sensory impairments, or physical disability, in children and adults. This is important to understand in view of the frequent coexistence of intellectual disabilities and autism. Sensory impairments and physical disability can impact upon communication, adding to the communication limitations a person experiences due to intellectual disabilities and/or autism. This adds complexity in differential diagnosis for clinicians when assessing other health conditions, and in their management. Hence, an understanding of the relationships between these impairments and conditions is needed to be able to assess the extent of their contribution in clinical presentations.

Studies have reported separately on the prevalence of sensory impairments and physical disability in people with autism and people with intellectual disabilities (as opposed to their relative contribution). Regarding people with autism, two large‐scale recent studies found blindness/partial sight loss in 12.1%, deafness/partial hearing loss in 14.1% and physical disability in 24.0% of autistic adults (Rydzewska et al., 2018a); and blindness/partial sight loss in 3.5%, deafness/partial hearing loss in 2.9% and physical disability in 10.7% of autistic children (Rydzewska et al., 2018b). These rates were much higher than those in the general population. In smaller studies, Gravel, Dunn, Lee, and Ellis (2006) found no significant differences on peripheral hearing in 37 children with autism, who were high functioning and did not present with significant cognitive or neurological deficits, compared to typically developing children (they did not report on central hearing impairment); Hewitt et al. (2012) reported visual impairments in 9.4%, hearing impairments in 5.7% and physical disability in 6.0% of 1,002 autistic adults, 90.3% of whom also had intellectual disabilities, as they were drawn from the population receiving intellectual and developmental disability services. These latter two studies are not representative of the population of adults with autism, nor of the whole population. These studies did not report on autistic people without intellectual disabilities, with the exception of Hewitt et al. (2012).

For people with intellectual disabilities, the reported prevalence rates for vision problems range from 4% to 99%, depending upon the populations studied and methods used (Janicki & Dalton, 1998; Kerr et al., 2003; van Splunder, Stilma, Bernsen, Arentz, & Evenhuis, 2003; Warburg, Rattleff, & Kreiner‐Moller, 1979) and for hearing impairments from 21% to 50% (Evenhuis, 1995; Evenhuis, Sjoukes, Koot, & Kooijman, 2009; Janicki & Dalton, 1998; Kinnear et al., 2018; Lavis, Cullen, & Roy, 1997; Yeates, 1992). A recent large‐scale population‐based study in Scotland reported vision impairment (47%) to be the most prevalent physical health condition reported in 1,023 adults with intellectual disabilities and hearing impairment the sixth most prevalent (27%) (Kinnear et al., 2018). A further, Scotland‐wide study of people with intellectual disabilities reported blindness/partial sight loss in 13.1%, deafness/partial hearing loss in 12.4% and physical disability in 32.6% (Hughes‐McCormack et al., 2017). These studies do not specify how many people with intellectual disabilities in their study also had autism.

There is considerably less research on individuals with coexisting intellectual disabilities and autism. Small‐scale studies include a study of 36 youth with co‐occurring intellectual disabilities and autism, age/gender matched with 36 people with intellectual disabilities without autism (Bradley & Bolton, 2006). They found that 38.9% with autism reported visual problems compared with 50.0% without autism, and 13.9% with autism reported hearing problems compared with 19.4% without autism. An intellectual disability register study reported that 95 of the 368 (25.8%) adults with intellectual disabilities and visual impairment also had markers for autism, compared with 422 of 2,674 (16%) of those with normal vision, and that 46 of the 60 (76.7%) of the adults with intellectual disabilities and congenital blindness also had markers for autism, compared with only 36 of the 67 (53.7%) with normal vision (Kiani et al., 2019).

The aforementioned studies reported intellectual disabilities and autism separately, or with the coexisting conditions. The present authors have been unable to identify any studies which have reported the relative extent to which being autistic or having intellectual disabilities accounts for the burden of sensory impairments or physical disability in the whole general population. This is therefore not clear. This is important to understand, given the frequent co‐occurrence of intellectual disabilities and autism.

The aim of this paper was to study the extent to which autism and intellectual disabilities are independently associated with sensory impairments and physical disability, in children and adults.

## 2 METHOD

### 2.1 Approval

Approval was gained from the Scottish Government for secondary analysis of the Scotland Census, 2011 data, and access to a subset of data was provided.

### 2.2 Census process and variables

The data source was Scotland's Census, 2011, which provides information on the number and characteristics of Scotland's populations on the Census day, 27 March 2011. The Census is undertaken every 10 years. It includes people both in private households and in communal establishments (such as care homes, registered group homes, prisons and student halls of residence). In private households, one person was responsible for completing the census details for all the household's residents; for communal establishments, the manager was responsible. Failure to provide information, or for providing false information attracted a fine of up to £1,000, for both people in private establishments and people in communal establishments, with no exemptions. Non‐responses were followed up by the Census team and help provided. Scotland's Census, 2011, achieved a 94% response rate (National Records of Scotland, 2013). The Census team adjusted for the 6% non‐response rate. This imputation process was conducted using a Census Coverage survey (including around 40,000 households) to estimate numbers and characteristics. The records from it were matched with Census records, to check for duplicates. Individuals estimated to be missing from the Census were then imputed, using a subset of characteristics from real individuals, including health information. This Edit and Imputation Methodology was adapted from the Office for National Statistics rigorous and systematic guidelines. Full details of the methodology and other background information on Scotland's Census, 2011, are available at http://www.scotlandscensus.gov.uk/supporting‐information.

The Census included questions on demography and long‐term conditions. The question on long‐term conditions asked the following: “Do you have any of the following conditions which have lasted, or are expected to last, at least 12 months? Tick all that apply.” Respondents were given a choice of 10 response options: (a) Deafness or partial hearing loss, (b) Blindness or partial sight loss, (c) Learning disability (e.g., Down syndrome), (d) Learning difficulty (e.g., dyslexia), (e) Developmental disorder (e.g., autistic spectrum disorder or Asperger's syndrome), (f) Physical disability, (g) Mental health condition, (h) Long‐term illness, disease or condition, (i) Other condition and (j) No condition. A write‐in box was also provided for respondents to report types of “other” condition/s.

For readers outwith Scotland, it should be noted that in Scotland, the term “learning disability” is synonymous with the international term “intellectual disabilities.”

The question on long‐term conditions was introduced into the Census for the first time in 2011 and included the question of autism spectrum conditions following lobbying by the Scottish autistic community. As part of the methodological preparations for Scotland's Census, 2011, as well as wide consultation on the question, the General Register Office for Scotland commissioned Ipsos MORI Scotland to undertake cognitive question testing of the question on long‐term conditions. This was to determine whether the questions were answered accurately and willingly by respondents, and what changes if any might be required to improve data quality and/or the acceptability of the questions. Cognitive interviewing is a widely used approach to critically evaluate survey questionnaires (Ryan, Gannon‐Slater, & Culbertson, 2012). It enables researchers to modify survey material to enhance clarity. Retrospective probing was deemed to be the most appropriate techniques, and this research was undertaken with 102 participants with a mix of gender and age, both with and without the health conditions and disabilities (including people with more than one of the conditions). This included people with autism, intellectual disabilities, dyslexia, dyspraxia, speech impairment, mental health conditions (both milder and more serious) and other long‐term conditions. The results found that the question on autism needed to be redesigned to that listed above, in order to more accurately capture the data specifically on autism. Additionally, the response “no” was amended to “no condition.” The questions on the other conditions did not require any modification. The cognitive question testing was important, in order to ensure language used was understood by, and in use by the whole lay Scottish population at the time, and correctly attracted the responses that were sought. If such a Census was undertaken in a different country, it is likely that the phrasing of the questions would need to be amended to accurately capture the language used and understood in that specific country at the time.

For 2.6% of the Census returns, information on long‐term conditions had not been completed. The Census team assumed the most plausible explanation was that the person had no long‐term condition but did not see the “no condition” checkbox at the end of the question, and hence recorded them to have none of the long‐term conditions.

### 2.3 Data analysis

Frequency data were generated followed by logistic regressions to calculate the odds ratios (OR) with 95% confidence intervals (CI) of autism, and intellectual disabilities (adjusted for age and gender) being associated with (a) blindness or partial sight loss, (b) deafness or partial hearing loss, and (c) physical disability. The gender variable was binary, and the reference group was male. The present authors conducted the analyses separately for children and young people (aged 0–24 years) and adults (aged 25+ years). This was because in Scotland's Census, 2011, the prevalence of autism is higher in the children and young people than in the adults, most likely due to widening out of the diagnostic criteria and greater awareness of autism in recent decades. Hence, the adults with autism are more likely to be on the more severely affected range of the autism spectrum. For the children and young people, the reference group was aged 0–15 years (childhood). The adults were grouped into 10‐year age‐bands, with the reference group being aged 25–34 years. The present authors then conducted a second round of the regressions, including the interaction terms age × intellectual disabilities, and age × autism. This was because the influence of age on impairments and disability is likely to differ in people with intellectual disabilities and possibly in people with autism to that seen in other people. All analyses were conducted with SPSS software version 22.

## 3 RESULTS

Scotland's Census, 2011, includes records on 5,295,403 people of whom 1,548,819 (29.2%) were children and young people, and 3,746,584 (70.8%) were adults aged 25 years and over. Of the children and young people, 9,396/1,548,819 (0.6%) reported having intellectual disabilities and 25,063/1,548,819 (1.6%) reported having autism. Of the adults aged 25 years and over, 16,953/3,746,584 (0.5%) reported having intellectual disabilities and 6,649/3,746,584 (0.2%) reported having autism. Of the children and young people with intellectual disabilities, 3,756/9,396 (40.0%) additionally had autism, and of the adults aged 25 years and over with intellectual disabilities, 1,953/16,953 (11.5%) additionally had autism. Of the children and young people with autism, 3,756/25,063 (15.0%) additionally had intellectual disabilities, and of the adults aged 25 years and over with autism, 1,953/6,649 (29.4%) additionally had intellectual disabilities.

### 3.1 Blindness/partial sight loss

10.9% of children and young people with intellectual disabilities but no autism, and 11.4% of adults with intellectual disabilities but no autism had blindness or partial sight loss. 1.5% of children and young people with autism but without intellectual disabilities, and 5.4% of adults with autism but without intellectual disabilities had blindness or partial sight loss. 0.4% of the children and young people with neither condition, and 3.1% of the adults with neither condition had blindness or partial sight loss.

Table 1 presents the OR (95% CI) of intellectual disabilities, and autism, adjusted for age, and gender, being independently associated with blindness or partial sight loss in the children and young people. It presents the results of two regressions, the second one also including adjustment for the interaction terms. Both intellectual disabilities (OR = 30.12) and autism (OR = 2.63) were independently associated with increased odds of having a blindness or partial sight loss, more so for intellectual disabilities. The association with blindness or partial sight loss was reduced by female gender (OR = 0.88) and increased by being a young person rather than a child (OR = 1.64).

**Table 1:** Odds ratios of autism and intellectual disabilities independently being associated with blindness in the whole population of children and young people, adjusted for age and sex

Variables	Regression 1		Regression 2 (including interaction terms)	
	Odds ratio	95% CI	Odds ratio	95% CI
Age				
0–15 (ref)	–		–	
16–24	1.49*	1.42–1.56	1.64*	1.56–1.73
Gender				
Male (ref)	–		–	
Female	0.88*	0.84–0.92	0.88*	0.84–0.92
Autism				
No (ref)	–		–	
Yes	2.45*	2.23–2.69	2.63*	2.32–2.96
Intellectual disabilities				
No (ref)	–		–	
Yes	23.52*	21.65–25.55	30.12*	26.91–33.70
Age × intellectual disabilities				
0–15 (ref)	–		–	
16–24	–		0.61*	0.51–0.72
Age × autism				
0–15 (ref)	–		–	
16–24	–		0.77*	0.63–0.93

* Statistically significant.

In adults (Table 2), a similar pattern was seen with both intellectual disabilities (OR = 16.89) and autism (OR = 3.29) being independently associated with blindness or partial sight loss; a reversed pattern was seen for female gender (OR = 1.02). All age groups had higher odds ratios than the 25‐ to 34‐year‐olds for association with blindness or partial sight loss, with a progressive gradient across the age groups.

**Table 2:** Odds ratios of autism and intellectual disabilities independently being associated with blindness in the whole population of adults, adjusted for age and sex

Variables	Regression 1		Regression 2 (including interaction terms)	
	Odds ratio	95% CI	Odds ratio	95% CI
Age				
25–34 (ref)	–		–	
35–44	1.36*	1.31–1.41	1.40*	1.34–1.46
45–54	2.33*	2.25–2.41	2.43*	2.34–2.52
55–64	3.89*	3.76–4.03	4.12*	3.97–4.27
65+	15.96*	15.48–16.47	16.98*	16.44–17.54
Gender				
Male (ref)	–		–	
Female	1.02*	1.01–1.03	1.02*	1.01–1.03
Autism				
No (ref)	–		–	
Yes	3.40*	3.61–4.30	3.29*	2.73–3.98
Intellectual disabilities				
No (ref)			–	
Yes	6.10*	5.80–6.42	16.89*	14.91–19.15
Age × intellectual disabilities				
25–34 (ref)	–		–	
35–44	–		0.67*	0.56–0.79
45–54	–		0.53*	0.45–0.62
55–64	–		0.35*	0.29–0.41
65+	–		0.13*	0.11–0.16
Age × autism				
25–34 (ref)	–		–	
35–44	–		1.06	0.81–1.40
45–54	–		1.03	0.79–1.32
55–64	–		1.03	0.77–1.38
65+	–		0.99	0.77–1.28

* Statistically significant.

### 3.2 Deafness/partial hearing loss

8.5% of children and young people with intellectual disabilities but no autism, and 12.6% of adults with intellectual disabilities but no autism had deafness or partial hearing loss. Two percent of children and young people with autism but without intellectual disabilities, and 9.3% of adults with autism but without intellectual disabilities had deafness or partial hearing loss. 0.6% of the children and young people with neither condition, and 9.1% of the other adults with neither condition had partial hearing loss.

Table 3 presents the OR (95% CI) of intellectual disabilities, and autism, adjusted for age, and gender, being independently associated with deafness or partial hearing loss in the children and young people. It presents the results of two regressions, the second one also including adjustment for the interaction terms. Both intellectual disabilities (OR = 13.98) and autism (OR = 2.31) were independently associated with increased odds of having deafness or partial hearing loss, more so for intellectual disabilities. The association with deafness or partial hearing loss was also reduced by female gender (OR = 0.93) and increased by being a young person rather than a child (OR = 1.56).

**Table 3:** Odds ratios of autism and intellectual disabilities independently being associated with deafness in the whole population of children and young people, adjusted for age and sex

Variables	Regression 1		Regression 2 (including interaction terms)	
	Odds ratio	95% CI	Odds ratio	95% CI
Age				
0–15 (ref)	–		–	
16–24	1.50*	1.45–1.56	1.56*	1.50–1.63
Gender				
Male (ref)	–		–	
Female	0.93*	0.90–0.97	0.93*	0.90–0.97
Autism				
No (ref)	–		–	
Yes	2.38*	2.17–2.60	2.31*	2.05–2.60
Intellectual disabilities				
No (ref)	–		–	
Yes	10.69*	9.80–11.67	13.98*	12.43–15.72
Age × intellectual disabilities				
0–15 (ref)	–		–	
16–24	–		0.57*	0.48–0.68
Age × autism				
0–15 (ref)	–		–	
16–24	–		1.02	0.85–1.23

* Statistically significant.

In adults (Table 4), a similar pattern was seen with both intellectual disabilities (OR = 7.47) and autism (OR = 2.36) being independently associated with deafness or partial hearing loss, with female gender lowering the odds (OR = 0.68). A gradient is seen, with the extent of association with deafness or partial hearing loss being progressively higher in older age groups.

**Table 4:** Odds ratios of autism and intellectual disabilities independently being associated with deafness in the whole population of adults, adjusted for age and sex

Variables	Regression 1		Regression 2 (including interaction terms)	
	Odds ratio	95% CI	Odds ratio	95% CI
Age				
25–34 (ref)	–		–	
35–44	1.77*	1.72–1.82	1.80*	1.75–1.85
45–54	3.55*	3.46–3.63	3.64*	3.55–3.73
55–64	7.75*	7.57–7.93	7.98*	7.79–8.18
65+	28.69*	28.06–29.34	29.62*	28.95–30.31
Gender				
Male (ref)	–		–	
Female	0.68*	0.68–0.69	0.68*	0.68–0.69
Autism				
No (ref)	–		–	
Yes	2.52*	2.32–2.73	2.36*	1.96–2.83
Intellectual disabilities				
No (ref)			–	
Yes	2.19*	2.08–2.29	7.47*	6.59–8.47
Age × intellectual disabilities				
25–34 (ref)	–		–	
35–44	–		0.55*	0.46–0.65
45–54	–		0.40*	0.34–0.46
55–64	–		0.27*	0.23–0.32
65+	–		0.13*	0.11–0.15
Age × autism				
25–34 (ref)	–		–	
35–44	–		0.90	0.69–1.17
45–54	–		0.94	0.73–1.21
55–64	–		0.91	0.71–1.19
65+	–		1.03	0.81–1.31

* Statistically significant.

### 3.3 Physical disability

30.6% of children and young people with intellectual disabilities, and 29.1% of adults with intellectual disabilities had physical disability. Five percent of children and young people with autism, and 14.4% of adults with autism had physical disability. 0.7% of the children and young people with neither condition, and 9% of the other adults with neither condition had physical disability.

Table 5 presents the OR (95% CI) of intellectual disabilities, and autism, adjusted for age, and gender being independently associated with physical disability in the children and young people. It presents the results of two regressions, the second one also including adjustment for the interaction terms. Both intellectual disabilities (OR = 43.72) and autism (OR = 5.62) were independently associated with increased odds of having physical disability, more so for intellectual disabilities. Physical disability was also associated with being a young person rather than a child (OR = 1.52), but gender was not associated.

**Table 5:** Odds ratios of autism and intellectual disabilities independently being associated with physical disability in the whole population of children and young people, adjusted for age and sex

Variables	Regression 1		Regression 2 (including interaction terms)	
	Odds ratio	95% CI	Odds ratio	95% CI
Age				
0–15 (ref)	–		–	
16–24	1.37*	1.33–1.42	1.52*	1.47–1.58
Gender				
Male (ref)	–		–	
Female	0.98	0.94–1.01	0.98	0.94–1.01
Autism				
No (ref)	–		–	
Yes	4.27*	4.01–4.54	5.62*	5.21–6.07
Intellectual disabilities				
No (ref)	–		–	
Yes	40.58*	38.39–42.90	43.72*	40.55–47.15
Age × intellectual disabilities				
0–15 (ref)	–		–	
16–24	–		0.88*	0.79–0.99
Age × autism				
0–15 (ref)	–		–	
16–24	–		0.45*	0.39–0.51

* Statistically significant.

In adults (Table 6), a similar pattern was seen with both intellectual disabilities (OR = 6.04) and autism (OR = 3.16) being independently associated with physical disability, as was female gender (OR = 1.07). A gradient is seen, with the extent of association with physical disability being progressively higher in older age groups.

**Table 6:** Odds ratios of autism and intellectual disabilities independently being associated with physical disability in the whole population of adults, adjusted for age and sex

Variables	Regression 1		Regression 2 (including interaction terms)	
	Odds ratio	95% CI	Odds ratio	95% CI
Age				
25–34 (ref)	–		–	
35–44	2.14*	2.09–2.19	2.14*	2.09–2.19
45–54	3.79*	3.71–3.87	3.79*	3.71–3.87
55–64	7.37*	7.22–7.52	7.37*	7.22–7.52
65+	15.51*	15.21–15.81	15.51*	15.21–15.81
Gender				
Male (ref)	–		–	
Female	1.07*	1.06–1.07	1.07*	1.06–1.07
Autism				
No (ref)	–		–	
Yes	3.16*	2.95–3.38	3.16*	2.95–3.38
Intellectual disabilities				
No (ref)	–		–	
Yes	6.04*	5.82–6.27	6.04*	5.82–6.27
Age × intellectual disabilities				
25–34 (ref)	–		–	
35–44	–		0.84	0.69–1.03
45–54	–		0.73*	0.60–0.90
55–64	–		0.71*	0.57–0.89
65+	–		0.72*	0.58–0.88
Age × autism				
25–34 (ref)	–		–	
35–44	–		0.45*	0.40–0.50
45–54	–		0.25*	0.23–0.28
55–64	–		0.16*	0.14–0.18
65+	–		0.09*	0.078–0.10

* Statistically significant.

## 4 DISCUSSION

### 4.1 Principle findings and interpretation

This is the largest, and possibly the only study of its type as far as the present authors are aware drawing from a whole‐country population, and is novel in taking into account the overlap between intellectual disabilities and autism unlike previous studies. The odds ratios for all of visual and hearing impairment and physical disability were much higher for both people with intellectual disabilities and people with autism, independently. So even taking into account that 20% had both intellectual disabilities and autism, autism still increased the odds of association with these three impairments/disabilities between 2.3 and 5.6 times, whilst having intellectual disabilities increased the odds to a greater extent; 6.0 to 43.7 times.

As our study is cross‐sectional, our findings do not indicate the direction of causation, or if there are other factors accounting for the associations between these impairments/disabilities and both intellectual disabilities and autism, which seems likely (Moreno‐de‐Luca et al., 2013). Our findings are important given the current gap in the literature on the relative independent size effects of associations of intellectual disabilities and autism with sensory impairments and physical disability, as clinicians need to have an increased awareness of potential co‐morbidities to ensure people with autism and people with intellectual disabilities have an improved quality of life.

### 4.2 Comparison with previous literature

No previous studies have been identified which investigate the extent to which autism and intellectual disabilities are independently associated with sensory impairments and physical disability in children and adults. The present authors believe this is therefore the first study to do so, and subsequently, the present authors cannot compare these results, until others have worked to replicate our findings.

There may well be several other confounders to our results that the present authors did not adjust for, but there is no existing literature to guide on these. It is therefore important that as well as studies to replicate our findings, future research should investigate other associations, particularly those that may be modifiable.

### 4.3 Strengths and limitations

The main strengths of the study are that it investigated the whole population in Scotland; it is one of the biggest population sizes to date (5.3 million); the conditions (intellectual disabilities, autism, visual and hearing impairments and physical disability) were also systematically enquired about for each person; and the phrasing of the questions underwent cognitive question testing in advance of the Census to ensure they captured the intended meaning. Consequently, the present authors believe these results are generalizable to other high‐income countries, as well as filling a significant gap in existing literature on the prevalence of impairments and disability in people with autism and intellectual disabilities separately.

Respondents reported whether or not each person had autism, intellectual disabilities and each of the sensory impairments and physical disability rather than each person receiving a clinical research assessment of these conditions (which would not be possible on such a large scale). This lack of validation means that reporting error is possible and has not been quantified. With regard to intellectual disabilities and autism, these conditions are typically diagnosed by multidisciplinary teams of professionals (including hearing assessments) during infant/primary school age, if not before, as in Scotland these diagnoses attract additional educational support which is to the child's advantage; once diagnosed, these are lifetime diagnoses. Consequently, there may be an undercount in the early years of childhood, whereas reporting of these conditions should be more accurate in later childhood, youth and adults, within the diagnostic criteria prevailing at the time of diagnosis. Additionally, differential diagnosis of intellectual disabilities or autism can be challenging, including taking account of similar repertoires of behaviours or symptoms due to other causes, such as sensory impairments. The proportion of people in the population reported to have autism was lower after age 25, reflecting the broadening of diagnostic criteria and greater awareness of autism in recent years; hence, the older people with autism might have more severe autism than the children/youth reported to have autism.

The present authors do not know the extent of whom self‐reported or for whom the report was by another person (e.g., parent); however, the latter is likely to be more common for the people with intellectual disabilities in view of their intellectual disabilities, and for the children and young people. Six percent of Census entries were imputed. Blindness and partial visual impairment were not differentiated, and nor was deafness from partial hearing impairment. This has the advantage of reducing reporting error, but has the disadvantage of producing broad‐brush rather than more detailed information. The Census did not enquire as to whether the impairments were congenital or acquired, or whether they were due to coexisting genetic syndromes. The Census team assumed the 2.6% who did not provide information on long‐term conditions did not have any of them, but the present authors are unable to confirm the accuracy of this assumption.

### 4.4 Implications

This study has provided information on the relative contribution of intellectual disabilities and autism on the population burden of sensory impairments and physical disability, and found both to be considerable. These have received more attention previously in people with intellectual disabilities than in people with autism. Our study now demonstrates that even when the coexistence of both intellectual disabilities and autism is taken into account, sensory impairments and physical disability are considerably more common in people with autism compared with other people, regardless of whether they also have intellectual disabilities. Awareness of the higher risk of sensory impairments and physical disability is important for clinicians, carers and family members to ensure identification in a timely manner in order to accurately plan for service provision and to tackle health inequalities. Additionally, sensory impairments and physical disability can impact upon communication, adding further to the communication limitations a person experiences due to their intellectual disabilities and/or autism. This adds further complexity for clinicians in the assessment of other health conditions, differential diagnoses and management of other health conditions. Clinicians need greater awareness of these additional complexities, and the associated reasonable adjustments they need to make to optimize communication, and to understand and correctly interpret knowledge to improve differential diagnosis and management. Hence, clinicians need to fully understand the relationships between these impairments and conditions to weigh up the extent of their contribution in clinical presentations. This appears to be highly important and so replication of our findings is needed through further research.

More evidence is needed to determine relationships between specific health conditions and associated factors among people with autism and intellectual disabilities separately. Doing so will influence the development of appropriate interventions, public health strategy and social care policy. Our study is robust and large scale but requires replication given the relative lack of previous research on this topic.

## CONFLICT OF INTEREST

None.
